# *Salvadora persica* mediated synthesis of silver nanoparticles and their antimicrobial efficacy

**DOI:** 10.1038/s41598-021-85584-w

**Published:** 2021-03-16

**Authors:** Hammad Arshad, Muhammad A. Sami, Saima Sadaf, Umer Hassan

**Affiliations:** 1 Institute of Biochemistry and Biotechnology, University of the Punjab, Lahore, Pakistan; 2 Department of Electrical and Computer Engineering, School of Engineering, Rutgers The State University of New Jersey, Piscataway, NJ USA; 3 Global Health Institute, Rutgers The State University of New Jersey, New Brunswick, NJ USA; 4 Department of Biology, Lahore Garrison University, Lahore, Pakistan

**Keywords:** Biotechnology, Nanobiotechnology, Nanoparticles

## Abstract

Silver nanoparticles (AgNPs) exhibit strong antimicrobial properties against many pathogens. Traditionally employed chemical methods for AgNPs synthesis are toxic for the environment. Here, we report a quicker, simpler, and environmentally benign process to synthesize AgNPs by using an aqueous ‘root extract’ of *Salvadora persica* (Sp) plant as a reducing agent. The synthesized *Salvadora persica* nano particles (SpNPs) showed significantly higher antimicrobial efficacy compared to earlier reported studies. We characterized SpNPs using UV–Vis spectroscopy, Fourier Transform Infrared Spectroscopy (FTIR), Transmission Electron Microscopy (TEM), Field Emission Scanning Electron Microscopy (FE-SEM), Dynamic Light Scattering (DLS) and X-ray powder diffraction (P-XRD). UV–Vis spectrum showed the highest absorbance at 420 nm. FTIR analysis depicts presence of bond stretching including OH– (3300 cm^−1^), C=N– (2100 cm^−1^) and NH– (1630 cm^−1^) which are attributed in the involvement of phenolics, proteins or nitrogenous compounds in reduction and stabilization of AgNPs. TEM, FE-SEM and DLS analysis revealed the spherical and rod nature of SpNPs and an average size of particles as 37.5 nm. XRD analysis showed the presence of the cubic structure of Ag which confirmed the synthesis of silver nanoparticles. To demonstrate antimicrobial efficacy, we evaluated SpNPs antimicrobial activity against two bacterial pathogens (*Escherichia coli* (ATCC 11229) and *Staphylococcus epidermidis* (ATCC 12228)). SpNPs showed a significantly high inhibition for both pathogens and minimum inhibitory concentration (MIC) and minimum bactericidal concentration (MBC) were found to be 0.39 µg/mL and 0.78 µg/mL for *E. coli* while 0.19 µg/mL and 0.39 µg/mL for *S. epidermidis* respectively. Further, Syto 16 staining of bacterial cells provided a supplemental confirmation of the antimicrobial efficacy as the bacterial cells treated with SpNPs stop to fluoresce compared to the untreated bacterial cells. Our highly potent SpNPs will likely have a great potential for many antimicrobial applications including wound healing, water purification, air filtering and other biomedical applications.

## Introduction

Nanotechnology has gained significant attention in the last few decades in many fields including medical, agriculture, food and cosmetics. Nanomaterials are ubiquitously used to kill microbial contaminants and finds applications in many areas of biomedical sciences^1–3^. Metal nanoparticles are currently being used in many areas including biosensors, imaging, drug delivery and nanomedicine^2,4^. In particular, silver nanoparticles (AgNPs) exhibit strong antifungal and anti-bacterial properties and are being explored for applications in water purification, textile industry, food packaging, cancer therapy, and wound dressing^2,5–8^. To synthesize AgNPs, a chemical reduction method is commonly used which requires highly toxic reducing chemicals such as sodium borohydride (NaBH_4_), polyethylene glycol (PEG), and sodium citrate (Na_3_C_6_H_5_O_7_) in aqueous or non-aqueous phase for Ag^+^ reduction^9^. Furthermore, for capping or stabilization of nanoparticles additional chemicals are further utilized including polyvinyl alcohol and polyethylene glycol^9,10^. These chemical methods lead to harmful chemical release in the environment and also make the synthesis process toxic as the byproducts of these chemical methods are hazardous for the ecosystem^10,11^.

Recently, NPs synthesis using biological resources has gained a significant attention to overcome the problem of non-ecofriendly chemical and toxic methods. Mainly, microbial and phyto mediated synthesis of NPs is reported in recent studies such as fungal mediated zirconium NPs for antimicrobial applications^12^ and plant mediated gold NPs for anticancer activity^13^. Similarly some of the earlier studies have used different plant extracts as a reducing agent in the AgNPs synthesis process^11,14^. Plant based synthesis has a major benefit over chemical methods since no hazardous chemicals are needed for the reduction and capping of AgNPs^11,15^. Various parts of plants such as leaves, bark, roots and fruit peels have been used in previous studies^5,14,16^. Additionally, many studies have reported AgNPs synthesis using agricultural waste^17^, enzymes^18^, insects’ metabolites^19^, and more interestingly animal wastes^20^ as well. Therefore, these biological materials for NPs synthesis are gaining dominancy over chemical or physical methods for AgNPs synthesis.

Traditionally, AgNPs have been extensively employed for antimicrobial applications due to their potent behavior against microorganisms^21–24^. Further applications of AgNPs include their usage as anti-tumor agent, to catalyze different pollutants (e.g. toxic dyes) and for water purification processes^7,11,25,26^.

Recent overuse of broad-spectrum antibiotics has led to a drastic increase in multi drug resistance (MDR) against microbial pathogens resulting in increased morbidity and costly care of patients. Many bacterial infections rely on antibiotics as a first cure and developing MDR will exacerbate their condition, this is especially true for critical care patients suffering from sepsis and other life-threatening conditions^27,28^. One of the mechanisms behind MDR pathogenesis is the production of β-lactamases, a family of enzymes which target β-lactam drugs including 4th generation cephalosporin and also compromise on many of the β-lactams^29^. Silver NPs have great potential to deal with MDR since no genotypic resistance is found in microbes for silver^30^. Therefore, AgNPs pose great potential to address the problem of MDR pathogens in clinical applications and have shown effective antimicrobial efficacy against a wide range of bacterial pathogens including gram-negative *Escherichia coli, Pseudomonas aeruginosa, Acinetobacter baumannii* and gram-positive *Staphylococcus* species etc. They also exhibit effective antimicrobial efficacy against fungal pathogens^31^. Recently, applications of AgNPs in other biomedical fields have also been reported, for instance, anticoagulant activity^32^, thrombolytic effect^33^, anti-filarial^34^, anti-leishmania^35^, antilarval, anthelmintic, antioxidant activity and anticancer^36^. The applications of AgNPs in biomedical and pharmaceuticals will continue to rise, owing to their potential industrial applications related to global health and eco-benign production requiring cheaper raw materials and cost-effective procedures.

Our current study is focused on synthesizing AgNPs using *Salvadora persica (S. persica)* which is a medicinal plant of the genus *Salvadora* widely found in South Africa and Asia^37^. This plant has been traditionally used for various ailments such as gastric troubles, sedative effects, anticonvulsant, antiulcer, etc. in different regions of the world^25,38^. Another common usage of this plant is for dental cleaning which has been used for centuries in some countries, a practice also supported by World Health Organization in customary areas^39^. An aqueous extract from *S. persica* has been used to produce AgNPs by reduction process and without requiring toxic reducing chemicals, hence providing an eco-friendly environment procedure and capping of AgNPs^14,16,25,40^.

Although inorganic nanoparticles found to have many applications in the biomedical field, however, they do pose certain disadvantages especially major limitation is related to genotoxicity properties of the NPs. Barabadi et. al. provided a comprehensive report addressing the issue of genotoxic profile of metallic NPs. Many studies reported a dose and time dependent genotoxic effect of the NPs regardless their mode of synthesis^41^. The main mechanism behind the genotoxicity of inorganic NPs is the production of ROS. Other mechanisms include the production of free radicals by NPs leading to redox (oxidation/reduction) reactions in the cell leading to apoptosis. The chronic oxidative stress also leads to the cell death which may be triggered by overexpressed inflammatory mediators (STATs and NF-kB), increased calcium levels and mitochondrial dysfunctions^42^. Therefore, a foremost issue of using the metallic NPs for humans/ animal trials will depend on future in-vivo studies as the toxicity profiles of inorganic NPs rely on many factors including synthesis parameters, type of biomaterials, quality of NPs etc.

A few reports earlier used different parts of *S. persica* to synthesize AgNPs, however, in this study, we used root extract and have demonstrated fewer manual processing steps to prepare plant extract for the synthesis process. Our SpNPs have shown significant increase in antimicrobial activity against targeted pathogens compared to other studies. We also performed an in-depth optimization of the synthesis process, determined antimicrobial efficacy of AgNPs against bacterial pathogens and studied the AgNPs effect by nuclear staining the bacteria. Our detailed synthesis and characterization give more insights into the effectiveness of the bio-reduction, stability, and size of the nanoparticles.

## Methods

### Synthesis of *S. persica* silver nanoparticles (SpNPs) (i.e. solution Z)

Salt of Silver nitrate (99.9%) was purchased from Sigma Aldrich, USA (209139-25G) and a stock of 0.1 M solution was prepared by mixing 1.69 g of silver nitrate in 100 mL of distilled H_2_O and stored in the dark (i.e. solution X). Further dilutions of 0.001 M, 0.005 M and 0.01 M were made from the stock solution by adding distilled H_2_O. All dilutions were stored in the fume hood and kept in dark for experiments. Roots of *S. persica* were purchased from Alkhair, Amazon (ASIN: B00IN6RSH6) and finely cut into small sections by using a sterile blade. The sections were thoroughly washed thrice with distilled H_2_O and subjected to drying at 50 °C in an incubator. A 20% w/v solution was prepared by adding 20 g of dried *S. persica* in 100 mL of distilled H_2_O. The mixture was heated and stirred for 15 min at 100 °C by using a digital magnetic hot plate stirrer at 500 RPM. The extract was centrifuged and filtered using a sterile syringe filter of 0.45 µm pore size (ASIN: B06Y15LBZ5) and kept in a closed sterile container (i.e., solution Y). Further dilutions were prepared to obtain a 10%, 5% and 1% of *S. persica* solution in sterile water and kept in a closed container. The protocol was validated by performing multiple times and the overall inspiration of the method was adopted from^31,43^.

In order to optimize the synthesis of *S. persica* silver nanoparticles (SpNPs), various concentrations of solutions X and Y were mixed together in 9:1 with final volume of 10 mL in sterile polypropylene vials. Initially 1 mM, 2 mM, 5 mM, 10 mM, 20 mM and 50 mM of solution X and 1%, 5%, 10% and 20% (w/v) of solution Y were used^44^. The vials were kept at room temp (25 °C) to observe yellow color development without any stirring in a dark place up to 96 h. The change in color was observed at 24 h interval and the yellow-colored mixtures were considered as positive reactions and were further subjected to different temperature and mixing conditions for optimized synthesis. For temperature study, vials were heated at 70 °C in an incubator and development of yellowish-brown color was observed after 24 h^31,40^. The similar reaction was carried out in glass vials on a magnetic hotplate stirrer at 200 RPM, and color change was monitored for 1, 2 and 4 h interval up to 24 h. Since the development of brown color is an indication of AgNPs synthesis^45^, the best vial was selected on the basis of short incubation time to develop brownish color. In order to study the effect of higher concentrations of *S. persica* extract on nanoparticles synthesis, the selected molarity and other conditions were kept constant, whereas the ratio of solution X to solution Y was increased as 8:2 and 7:3. The synthesized SpNPs were washed to remove impurities by centrifugation at 5000 RPM for 15 min followed by the removal of supernatant and addition of distilled H_2_O. The same step was repeated three times to get purified SpNPs suspended in distilled H_2_O. The final ratio was subjected to pH study. A 10 mM sodium hydroxide (NaOH) was used to change the pH of solution Y to 8, 9 and 10. Synthesized nanoparticles (solution Z) were characterized by monitoring changes in the brown color and UV–Vis analysis. We also took solution Z images using the camera and imported those in Image J for subsequent analysis. We found mean pixel intensity (MPI) of solution Z after each interval of time to quantify the change in color.

### Characterization

The synthesized eco-friendly *Salvadora persica* nano particles (SpNPs) were characterized in detail by using UV–Vis spectroscopy, FTIR, TEM, FE-SEM, DLS, and P-XRD. **UV–Vis spectroscopy**: Synthesized SpNPs were characterized by UV–Vis spectroscopy. The spectrum scan was carried out by using a single beam spectrophotometer (VWR UV-1600) attached to ‘Multi Wavelength Professional’ computer software. UV–Vis spectroscopy was carried out from 300–900 nm wavelength range. We used distilled H_2_O as a blank in all scans. **FTIR** was carried out by using a portable 4300 handheld spectrometer by Agilent. Briefly, 50 µL of the liquid sample was placed on the diamond attenuated total reflectance (ATR) assembly and analyzed. The analysis was carried out for solution Y and solution Z in triplicates. **TEM** was performed on a Philips CM12 with AMT-XR11 digital camera and images were collected at 80 kV acceleration. The samples for TEM were prepared by placing a drop of SpNPs (10 µg/mL) sample on a copper grid containing carbon film (VWR, 100503-154) and dried overnight in desiccator at room temperature. **SEM and DLS**: The morphology of NPs was observed by FE-SEM, Zeiss Sigma, Model 8100. Sample preparation was carried out by diluting SpNPs in distilled H_2_O at a concentration of 0.1 µg/µL followed by drop-casting on a silicon wafer (10 mm) which was attached with aluminum stubs (16111, Ted Pella, Inc.) using carbon tape (16084-1 PELCO Tabs). Samples were analyzed using 5–10 kV. Size distribution of the SpNPs was carried out using Malvern Zetasizer Ultra based on DLS using plastic disposable cuvettes. The data of the scan was further analyzed using Origin software (OriginLab Corporation). **P-XRD**: The analysis was obtained by using a Bruker Vantec-500 area detector and a Bruker FR571 (Cu Ka; l = 1.5418 Å). Data was collected at room temperature (25 °C) with a sample to detector distance of 10 cm. The range for XRD scan was from 20 to 60 with ω scan of 2 degrees.

### Antimicrobial assay

Gram-negative *Escherichia coli* ATCC 11229 (Microbiologics, Catalog No. 0681A) and gram-positive *Staphylococcus epidermidis* ATCC 12228 (Microbiologics, Catalog No. 0371P) were used for the antimicrobial studies. Bacterial lyophilized pellets were cultured on nutrient agar plates according to the user’s manual instructions^46^. The antimicrobial activity testing was carried out by using Kirby Bauer susceptibility method^47^ using 0.5 mm discs (Z7121, Hardy Diagnostics) loaded with 20 µL of solution X and Y each, 20 µg and 40 µg of solution Z respectively on nutrient agar plates (Evviva Sciences ASIN: B07CSKBD8T) containing bacterial inoculum. The same approach was used for the agar well method. The wells were made in agar by using a pipette tip of 0.5 cm in diameter under sterile conditions. Chloramphenicol discs, 30 µg (Z8341, Hardy Diagnostics) having 0.5 cm diameter were used as a positive control in all antimicrobial experiments. Test strains were uniformly spread using sterile cotton swabs on the agar plates. The OD_600_ nm was measured to be 0.1 representing concentration of ~ 1 × 10^7^ CFU/mL for both strains in phosphate buffer saline (PBS). The test samples were introduced into blank discs and agar wells and left for 10 min in order to adsorb in the agar medium. Agar plates were placed in an incubator at 37 °C for 24 h. Subsequently, the zones of inhibition were measured around each test sample in mm and recorded.

### MIC and MBC

MIC and MBC were found by using broth assay^48^ . SpNPs were serially diluted to make final concentrations from 0.19 µg/mL to 25 µg/mL in Nutrient broth in 24 well plates. Overnight grown bacterial culture was diluted in PBS to obtain a concentration of ~ 1 × 10^7^ CFU/mL (which is monitored as OD_600_ nm = 0.1) and a 2 µL of culture was inoculated in each well except blanks and incubated at 37 °C for 24 h. Bacteria without SpNPs were used as a negative control whereas blank wells contained nutrient broth only. OD_600_ nm values were determined after 24 h followed by the CFU count on agar plates. The minimum concentration of solution Z which led to 98% reduction in CFU as compared to positive control was noted as MIC. The next higher concentration of solution Z was confirmed as MBC, since there was 100% reduction in CFU. All samples were tested in triplicates under aseptic conditions.

### Nuclear staining of SpNPs treated microbial cells

Freshly cultured *E.coli* and *S. epidermidis* were suspended in PBS to make OD_600_ as 0.1. This is followed by treatment with SpNPs (1, 100 and 500 µg/mL) for 4 h. A 5 µM Syto 16 (Invitrogen, Catalog number: S7578, i.e., a green fluorescent nucleic acid stain) was used to analyze bacterial cells with SpNPs and without (control). Bacterial cells were gentle mixed with Syto16 in 1:1 and kept in dark for 15 min at room temperature. The cells were placed on a glass slide with a coverslip and observed under Olympus IX81 fluorescent microscope using a filter with 465–495 nm excitation and 515–555 nm emission spectrum. The protocol was optimized after multiple experiments and inspired by^49,50^.

## Results

### Synthesis

**Figure 1:**
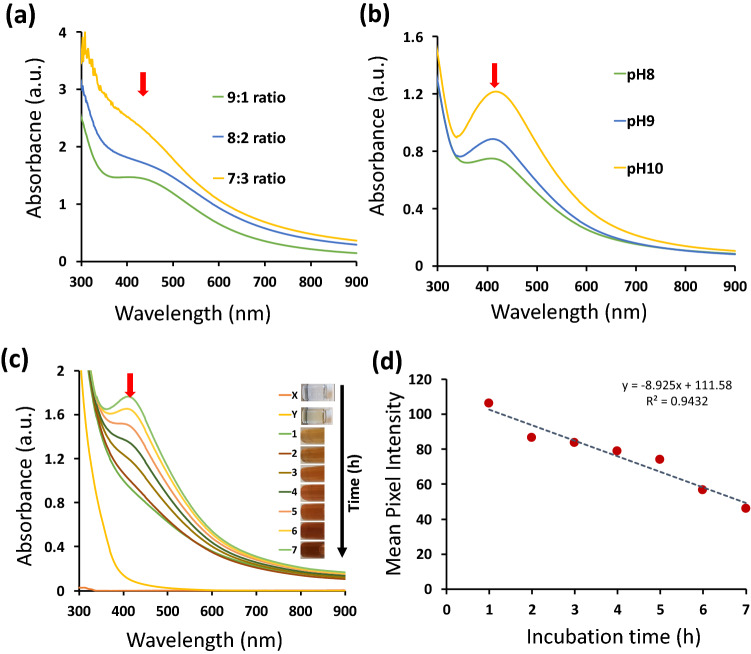
UV–Vis spectrum (black arrows shows peak absorbance at 420 nm in the spectrum). The ratio of solution X to Y was optimized as indicated in (**a**) where the 9:1 shows the higher absorbance. The effect of pH on SpNPs synthesis was also observed and shown in (**b**), the rise in pH indicated better synthesis. Detailed time-based synthesis of solution Z is shown in (**c**) which confirmed the formation of SpNPs after 3 h. No absorbance is resulted for solution X and Y as shown by UV–Vis spectrum in (**c**), Mean pixel intensity (MPI) plot (**d**). The change in color vs. incubation time has shown for solution Z. The decrease in MPI revealed the increase of color darkness.

The synthesis for quick and stable SpNPs was optimized as shown in Table S1 and Fig. S1. The nanoparticles were successfully synthesized by using 10% w/v *S. persica* aqueous extract (solution Y) and 10 mM solution of silver nitrate (solution X) with continuous stirring and heating at 70 °C. The 9:1 ratio of solution X to Y was found to be best in order to synthesize stable and dispersed SpNPs. The change in color was observed after 60 min with gradual increase up to 7 h of incubation time, which showed a clear growth of SpNPs as shown in Fig. 1. The synthesized SpNPs (solution Z) solution changed its color from clear to dark brown as shown in Fig. S2 (a). Further, the 20% solution Y with 9:1 also resulted in formations of SpNPs but with more aggregation as shown in Fig. S2 (b). It was noticed that the alkaline solution Y at pH 10 produced the SpNPs with greater dispersion as shown in Fig. 1. Variation in the pH of solution Y (pH: 8–10) influenced the synthesis process as shown in the Fig. 1b. A higher absorbance value was observed which represents smoother bio-reduction of Ag^+^ to Ag^0^ that lead to quicker and stable synthesis of the AgNPs. The use of 20 mM and 50 mM solutions also resulted in nanoparticles formation with higher agglomeration as shown in Fig. S2 (b).

### Characterization

Synthesis optimization including the selection of solution Y concentration and pH was performed by the UV–Vis spectrum as shown in Fig. 1(a,b). The maximum absorbance of SpNPs between 400–450 nm is pointed by arrows in the Fig. 1. Further, no peak was observed for solution X and solution Y in the same region as expected which confirms the presence of silver nanoparticles only in solution Z (Fig. 1c). The time-based spectrum confirmed the synthesis of SpNPs after 3 h of incubation, however, the best result was observed after 7 h achieving highest absorbance at 420 nm as shown in Fig. 1c. The synthesized SpNPs (solution Z) solution changed its color from clear to dark brown as shown in Fig. 1c which was also confirmed by MPI analysis as shown in Fig. 1d. It shows an interesting relation of a linear decrease in MPI as the incubation time in increased from 1 to 7 h (R^2^ = 0.94).

**Figure 2:**
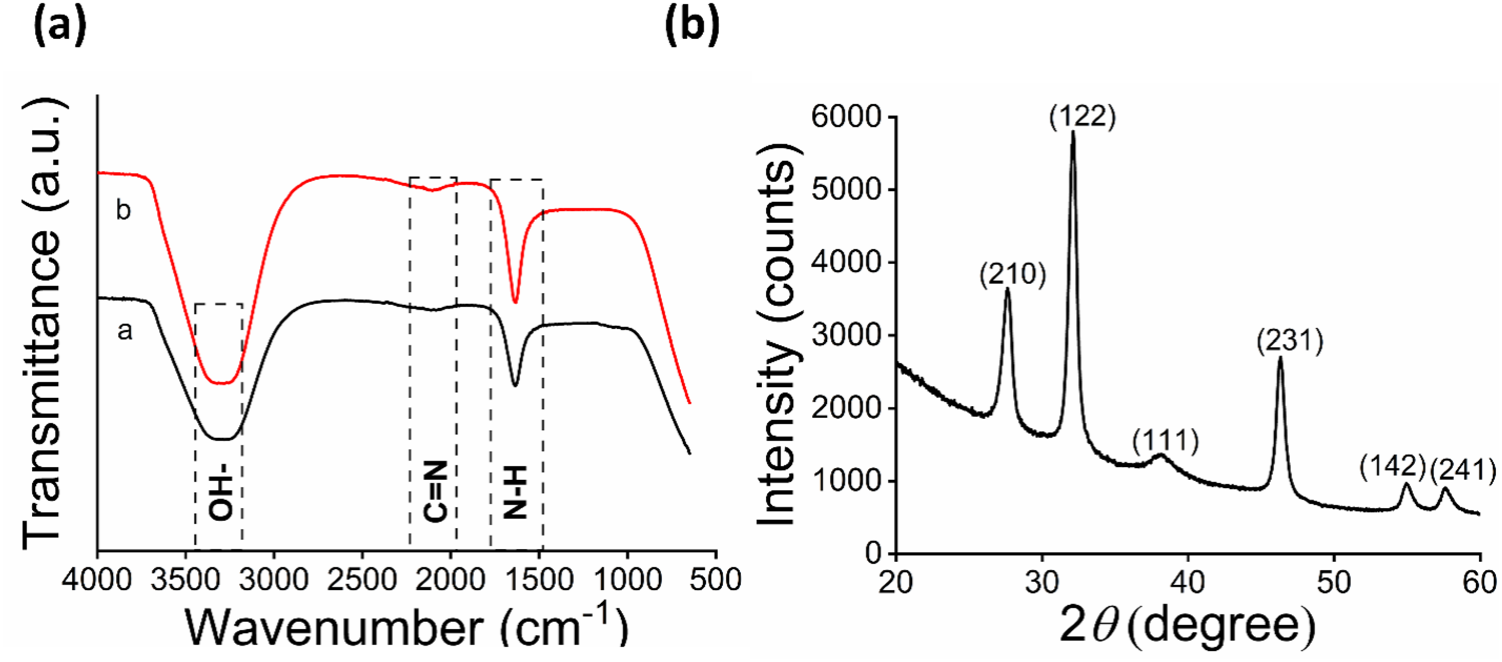
Characterization of SpNPs. FTIR analysis for phytochemicals involvement is shown in (**a**), where (a) represents *S. persica* extract and (b) SpNPs. Powdered XRD analysis is depicted by (**b**) and shows 2θ values for SpNPs.

FTIR analysis of SpNPs and solution Y suspended in distilled H_2_O (Fig. 2a) revealed the presence of certain bond stretching including OH– stretching (3300 cm^−1^), C=N– stretching (2100 cm^−1^) and NH– (1630 cm^−1^). In particular, bond stretching for OH– depicts the involvement of phenolic compounds such as tannins, saponins, flavonoids. This in in support of earlier studies demonstrating OH– bond stretching in relation to AgNPs synthesis by^39,51^. Similarly, C=N and N–H stretches showed the presence of amino acids, peptides or nitrogen containing aromatic compounds such as aniline, benzylamine etc. Similar role of phytochemicals has been found by^39,52^. Note that Ag^+^ have been reduced to Ag^0^ by the addition of electrons coming from the reducing phytochemicals such as proteins and phenolics present in solution Y. The comparison of SpNPs (Fig. 2a) and plant extract (Fig. 2b) seems to have similar head groups (OH–, NH– etc.) confirming the involvement of *S. perisca* in reduction and stabilization of SpNPs. The analysis of powdered XRD show distinctive diffraction peaks at 2θ = 27.69°, 32°, 38.1, 46.0°, 55°, and 57.6° as shown in Fig. 2b which corelates to the following lattice planes i.e. (210) (122) (111) (231) (142) and (241). This confirmed the lattice planes of FCC crystal structure of SpNPs. Similar patterns have also been reported in other studies^25,43^.

**Figure 3:**
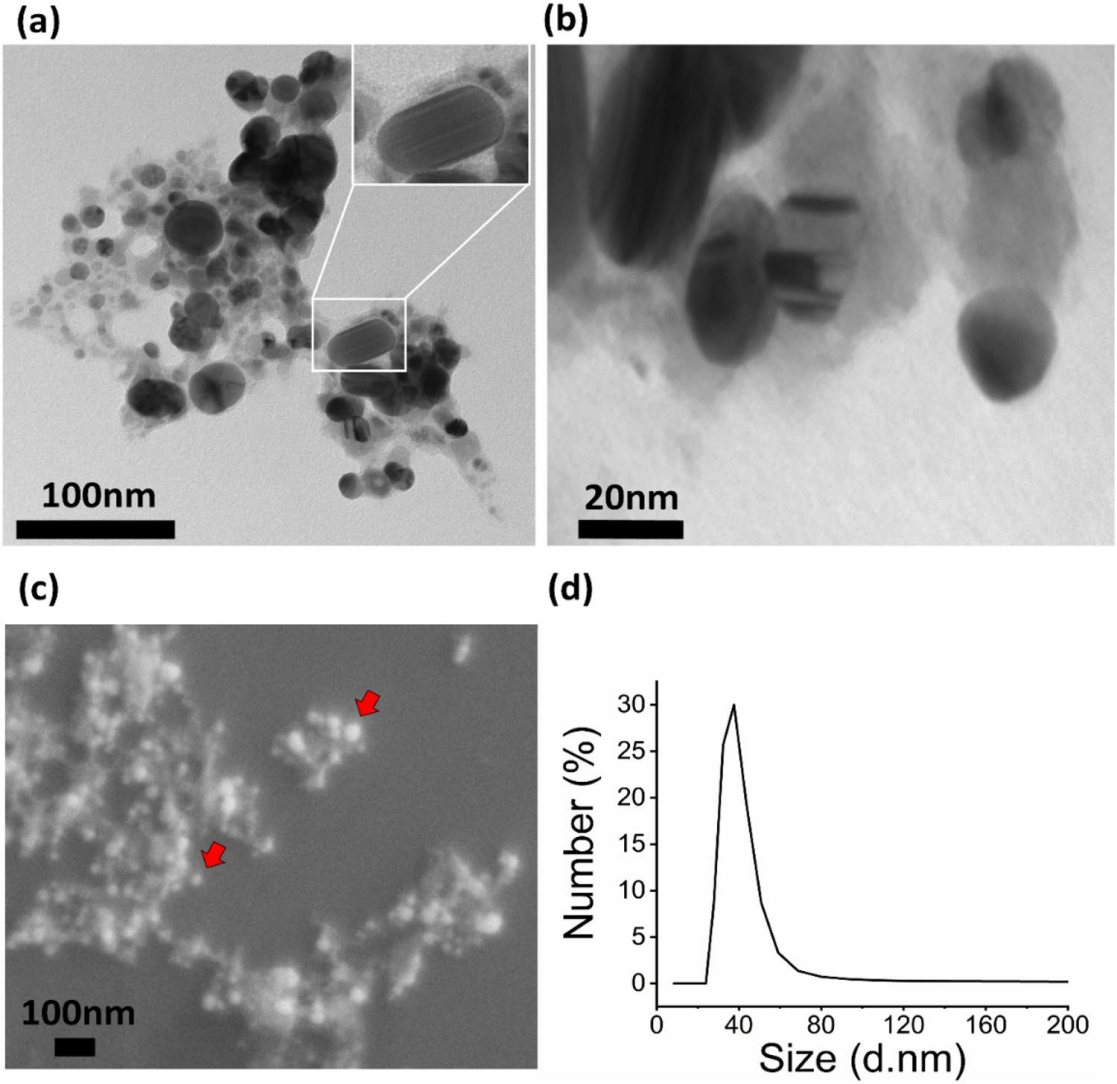
Size and morphology study. TEM analysis shows the formation of spherical and rod shaped (inset) SpNPs of size range of 10—70 nm (**a**,**b**), further analysis with FE-SEM depicted the presence of spherical nanoparticles as indicated with red arrows in (**c**) while DLS also show similar average size of 37.5 nm of synthesized SpNPs (**d**).

Figure 3(a,b) illustrates the morphology and size distribution of SpNPs under TEM. The formation of nano-spheres with an average size of 35–40 nm is confirmed while a small percentage of nano rods were also observed as shown in inset of Fig. 3(a). These nano spheres and nano rods showed good dispersion with less aggregation. *S. persica* exhibits a role of strong sticky material which can be observed in Fig. 3(a,b). FE-SEM analysis shown in Fig. 3c also confirms the spherical and rod shape of SpNPs. The overall dispersion pattern of FE-SEM is like the TEM images. Finally, we also performed DLS analysis which again confirmed the size distribution of SpNPs to be 37.5 nm on average as shown in Fig. 3d.

### Antimicrobial assay

**Figure 4:**
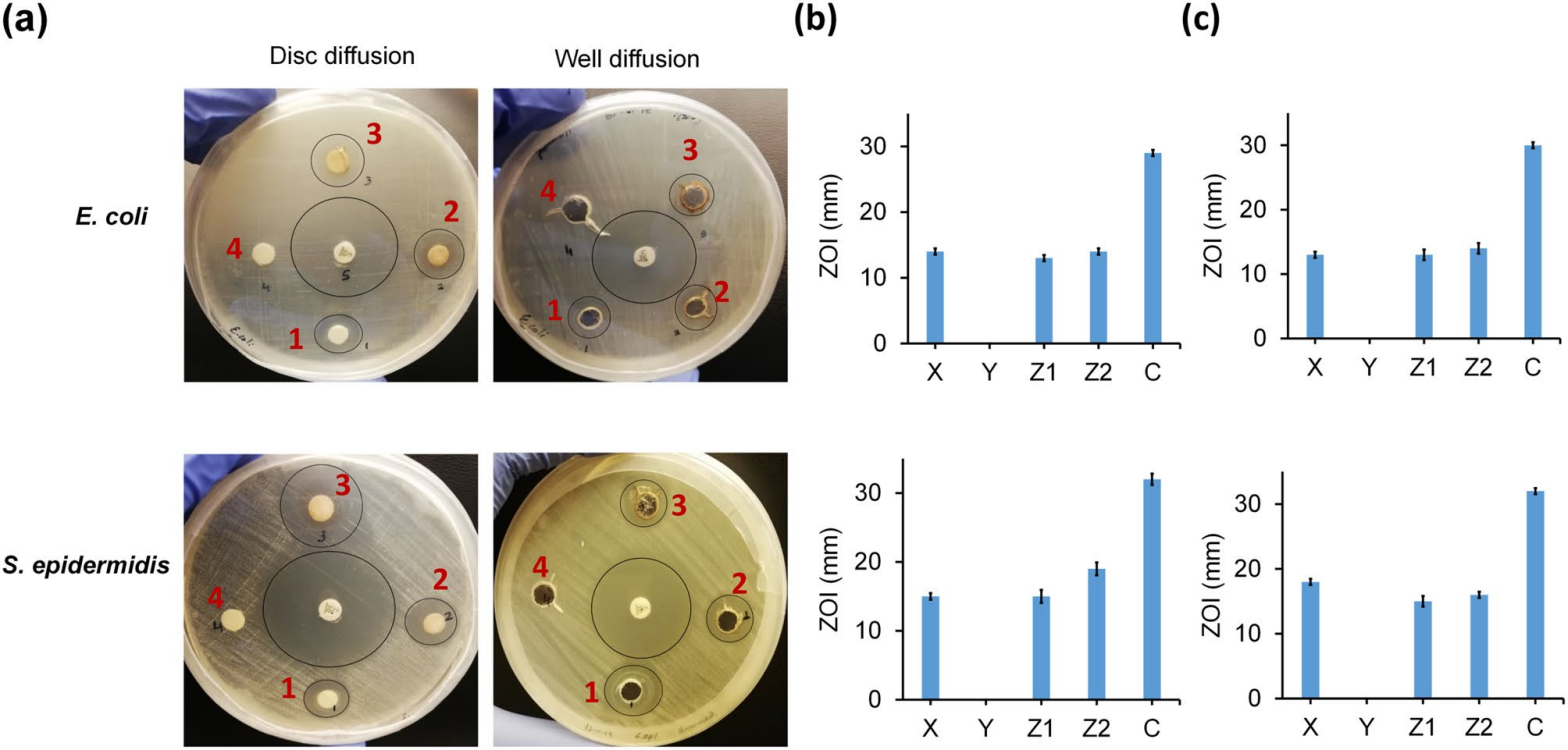
Antibacterial effect of SpNPs on *E. coli* and *S. epidermidis*. Antimicrobial analysis was performed by using disc diffusion and well diffusion methods on nutrient agar plates (**a**). Four samples were used i.e. (1) 20 µl of AgNO_3_ (10 mM), (2) 20 µg SpNPs (Z1), (3) 40 µg SpNPs (Z2) and (4) 20 µl of *S. persica* (10%) extract. Chloramphenicol was used as a positive control in all experiments in the center of the well plate. Inhibition zones around samples are encircled. The zones of inhibition were calculated by using a scale in mm and their mean value is represented by bar plots and standard deviation as error bars. ZOI for disc diffusion method for all samples are shown in (**b**) and ZOI for well diffusion are shown in (**c**). All experiments were performed in triplicates.

We characterized the antimicrobial effect of SpNPs for gram-negative *E. coli* (ATCC 11229) and gram-positive *S. epidermidis* (ATCC 12228) and found to be highly effective against both bacterial strains. The zones of inhibition (ZOI) for 20 µL of AgNO_3_ (10 mM), 20 µg of SpNPs, 40 µg of SpNPs, and 20 µL of *S. persica* (10%) extract are labelled as 1, 2, 3, and 4 respectively in Fig. 4a. Chloramphenicol is used as a positive control and its ZOI is shown in the center of each well plate. The SpNPs at 20 µg and 40 µg (shown as Z1 and Z2 respectively) were effective and produced significant ZOI in both well diffusion and disc diffusion methods. Figure 4b shows the bar plots representing the mean and standard deviation values for ZOI in disc diffusion method. ZOI produced by solution X, solution Y, solution Z1, solution Z2 and chloramphenicol were 14.33 ± 0.58 mm, 0 ± 0 mm, 12.67 ± 0.58 mm, 14.33 ± 0.58 mm and 29.33 ± 0.58 mm against *E. coli* while 15.33 ± 0.58 mm, 0 ± 0 mm, 15.33 ± 1.15 mm, 18.67 ± 1.15 mm and 32.00 ± 1.00 mm against *S. epidermidis* respectively. Similarly, Fig. 4c shows the bar plots representing the mean and standard deviation values for ZOI in well diffusion method. ZOI produced by solution X, solution Y, solution Z1, solution Z2 and chloramphenicol were 13.33 ± 0.58 mm, 0 ± 0 mm, 13 ± 1 mm, 14 ± 1 mm and 29.68 ± 0.58 mm against *E. coli* while 17.67 ± 0.58 mm, 0 ± 0 mm, 15 ± 1 mm, 15.67 ± 0.58 mm and 31.67 ± 0.58 mm against *S. epidermidis* respectively. We did not observe any ZOI for solution Y. The MIC and MBC assay was carried out using broth assay and values were found to be 0.39 µg/mL and 0.78 µg/mL for *E. coli* whereas 0.19 µg/mL and 0.39 µg/mL for *S. epidermidis* respectively.

### Nuclear staining of bacterial cells

**Figure 5:**
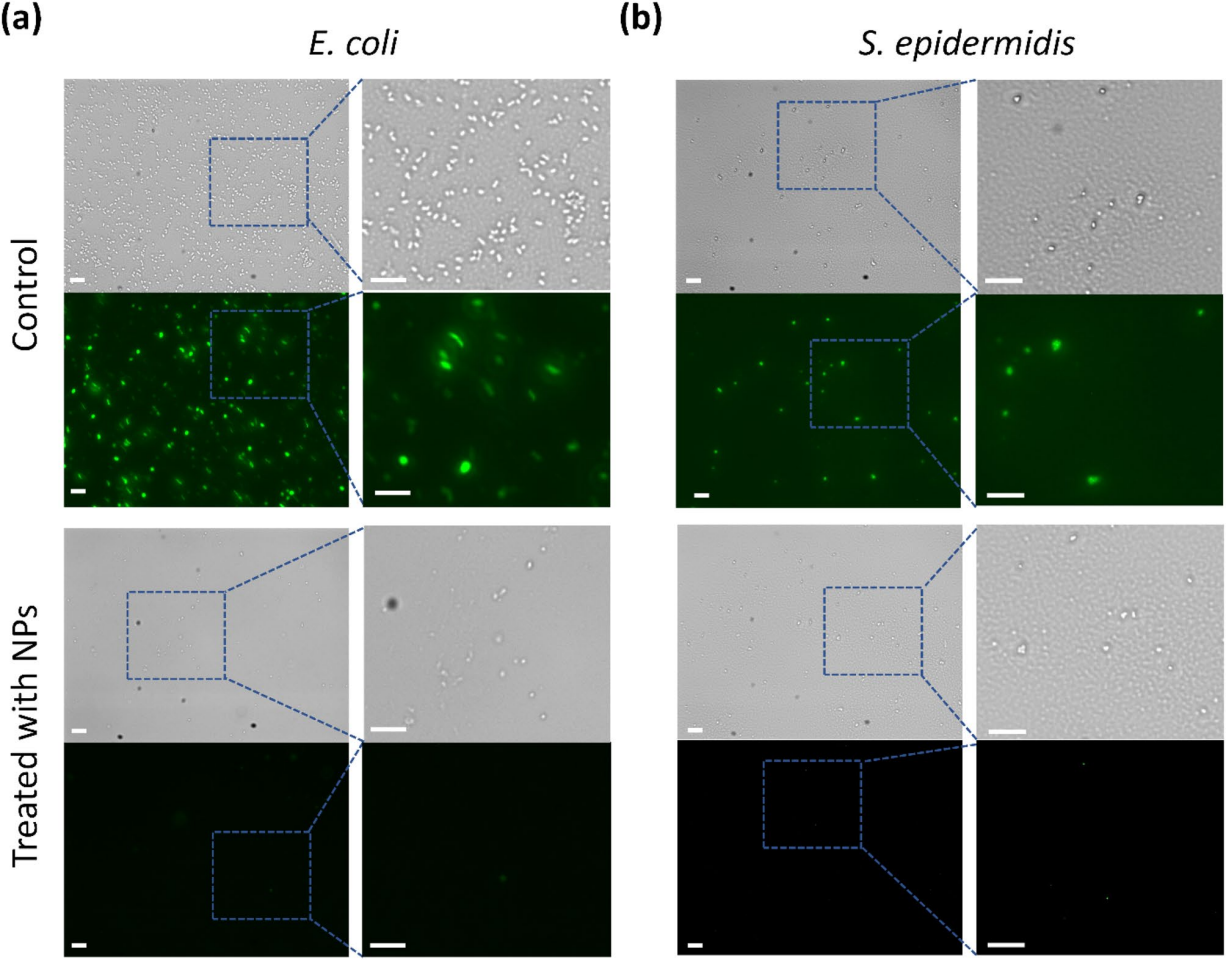
Microbial cell viability. *E. coli* (**a**) and *S. epidermidis* (**b**) were observed using green, fluorescent nuclear stain, Syto 16*.* The comparative analysis is shown between bacterial cells with (treated with SpNPs) and without the treatment of SpNPs (control). The treated cells exhibited negligible fluorescence as compared to the untreated microbial cells. Bright field and fluorescent images are shown on up and down positions, respectively. Selected areas are zoomed-in for better microbial cell visualization. The scale bar is 10 µm for all images.

*E. coli* (Fig. 5a) and *S. epidermidis* (Fig. 5b) were stained by using a green fluorescent stain, Syto 16. Figure 5 shows both bright field (left) and fluorescent images (right). The cells without the treatment of SpNPs (control) showed the fluorescence while the treated *E. coli* and *S. epidermidis* did not produce any fluorescence.

## Discussion

The synthesis process was monitored in detail and bio-reduction of Ag + to Ag^0^ was achieved using *S. perisca* aqueous root extract which was initially observed by the color change from yellow to brown (Fig. S1). This phenomenon is due to surface plasmon resonance (SPR) of AgNPs as previously described by many studies utilizing either green or chemical methods for silver nanoparticles synthesis^40,45^. In this study, the changes in color starts from 1 h of incubation whereas the absorbance peak started at 3 h of incubation which presents the bioreduction of Ag^+^ with the passage of time. To the best of our knowledge, there is one more study which used *S. perisca* roots for AgNPs synthesis^40^. Our study demonstrated simpler method compared to earlier studies which reported multistep extraction processes for preparing *S. persica* extract and rely on vacuum based evaporation procedure that is more laborious and time consuming^40^.

Characterization using UV–Vis spectrum is commonly used to confirm the presence of AgNPs in a solution and the absorbance between 400–450 nm is measured. In our study this was evident from UV–Vis spectrum that the SPR was visible between 400 to 450 nm and the highest excitation of AgNPs was at 420 nm (Fig. 1) due to vibrational movement of electrons at that wavelength depending on size and shape of SpNPs which is in line with other related studies^2,5,14,36^. Further, we observed that a higher concentration of the plant material (solution Y) to silver nitrate (solution X) exhibit a direct effect on the size and dispersion of the SpNPs. We observed this phenomenon by color change, UV–Vis spectrum, and DLS analysis (Fig. S1-S3 and Fig. 1c). The lower amount of plant extracts is known to synthesize smaller sized particles, which is also validated in our study. Our nanoparticles aggregation analysis revealed that using higher concentrations of solution X resulted in the agglomerated nanoparticles which directly influenced the UV–Vis spectrum and the broader spectrum peaks with noise represent agglomeration of AgNPs (Fig. S2). Here, we also found that higher alkaline pH values resulted in faster particle synthesis. One possible reason could be higher pH likely provide more electrons in the reaction mixture which facilitates overall reduction process.

In plant-based synthesis process, phytochemicals are involved in the reduction and capping of the AgNPs. Our FTIR analysis depicted the involvement of hydrophilic phytochemicals present in *S. persica* extract such as sugars (–OH), proteins and amines (NH–) or other phytochemicals (C=N–) in the biosynthesis and stabilization of SpNPs. FTIR analysis of SpNPs was similar when compared to solution Y as shown in Fig. 2a. The presence of the head groups in the SpNPs confirmed the involvement of solution Y as also evident in previous studies^40,48^. Furthermore, our TEM and FE-SEM analysis illustrated the spherical and rod nature of SpNPs as shown in Fig. 3 and have shown least aggregation of the SpNPs compared to most of the previous reports^31,53,54^. Our method produced a heterogeneous distribution of nanoparticles representing morphological variation. The presence of *S. persica* is known to hold the AgNPs and other nanoparticles which is also visible in our study (Fig. 3b)^16^. This confirms the role of *S. persica* as reducing and stabilizing agent. Our SpNPs size range was variable and zeta sizer analysis depicted the average size as 37.5 nm. We also found that SpNPs remained stable and dispersed after many weeks possibly because of the improved processing including washing steps to remove excessive reactants and impurities.

Table 1 provides a comparative analysis of our study with other studies utilizing *S. persica* (different parts of plant) for AgNPs synthesis. Our method demonstrates a simpler synthesis process, and much higher antimicrobial efficacy against pathogens. Antimicrobial efficacy of our SpNPs was significantly higher for both bacterial strains (gram –ve and + ve) as compared to another comparative study (using roots for particle synthesis). Shaik et.al. used 100 µg of SpNPs to produce comparable ZOI to our study which requires only 40 µg SpNPs. Other studies which used *S. persica* leaves for particle synthesis; reported MIC and MBC for gram-negative and gram-positive bacteria to be 100 µg/mL and 200–400 µg/mL respectively which is 100 to 400 times higher compared to our reported value (< 1 µg/mL).

**Table 1:** The comparative analysis of current and previous studies. The characteristics of SpNPs produced by *S. persica* has been shown. Present study presented simpler method, lesser MIC and MBC and live dead cell analysis to understand mechanism of action on bacterial pathogens. N/A: Not available.

	Current study	^34^	^23^	^12^	^40^
Synthesis process	Heating and mixing 3–7 h at 70 °C	Reflux and vacuumed and heating at 90 °C	Heating at 120 °C and mixing	Room Temperature 1 h	Room Temperature in dark – 24 h
Plant material	*S. persica* roots	*S. persica* roots	*S. persica* stem	*S. persica* bark	*S. persica* leaves
Size of NPs	10–70 nm (average is 37.5 nm	10–20 nm	1–6 nm	50 nm	3–5 nm
Shape of NPs	Spherical and rod	Spherical	Spherical	Spherical	Spherical
Characterization	UV–Vis, FE-SEM, TEM, XRD, FTIR, DLS	UV–Vis, SEM, HR-TEM, XRD, FTIR, EDX	UV–Vis, SEM, HR-TEM, XRD, FTIR, EDX	UV–Vis, SEM, HR-TEM, PXRD	UV–Vis, FTIR TEM, SAED, XRD
MIC and MBC (µg/ml)	0.19 and 0.39 for *S. epidermidis (ATCC 12228)* 0.78 and 0.39 for *E. coli (ATCC 11229)*	N/A	N/A	200 and ≥ 400 for *S. aureus (ATCC 25923)* 100 and 200 for *E. coli (ATCC 25,922)*	N/A
ZOI (mm)	20 for *S. epidermidis* and 15 for *E. coli* at 40 µg/ well	15 for *S. aureus* (ATCC 29213) and 13 for *E. coli (ATCC 25922)* at 100 µg/ well *Pseudomonas aeruginosa* (ATCC 27853) *Micrococcus luteus* (ATCC 10240)	N/A	12 for *S. aureus* and 13 for *E. coli* at 10 µl/disc	N/A
Pathogens used	2 (Gram-positive and Gram-negative)	4 (Gram-positive and Gram-negative)	N/A	2 (Gram-positive and Gram-negative)	N/A
Cell staining validation	Yes	N/A	N/A	N/A	N/A

We also observed that our solution Y did not produce any ZOI in both strains which is contrary to the work done on *S. persica* leave extract^48^ and likely because of using diluted *S. persica* extract (10% w/v or 100 mg/mL) in our study; as higher concentration is required for inhibition^55^.

The biological activity of inorganic NPs such as silver, gold, selenium etc. is mostly influenced by a variety of factors including size distribution, morphology, capping agents, aggregation etc.^56–58^. Many studies have shown that the biological activity such as antineoplastic and antimicrobial efficacy of biogenic metallic NPs are variable and likely because of the variation in biomolecules present for bio reduction and capping of NPs^57^. Shape and size have direct influences on the NPs efficiency for example, it has been shown that the rod-shaped gold NPs poorly penetrates into cancer cells as compare to spherical NPs. Smaller NPs are also found to be more cytotoxic to normal and cancer cells as compared to larger particles and on the other hand are more effective against microbial pathogens. Moreover, it is also reported that positively charged surface NPs has higher tendency to bind with mammalian cells due to negatively charged membranes. Other factors such as surface chemistry and capping agents of NPs depends on the type of conjugated biomolecules, for instance the attachment of alkaloids, phenolics, enzymes, proteins or sugars change the behavior of NPs. The variety of fabrication of NPs leads not only to versatile functions of NPs but also different consideration for with respect to their applications. For example, drug delivery application may require the attachment of certain antibodies, aptamers etc. to NPs^56,57,59^.

The overall antimicrobial performance of SpNPs was observed to be better in comparison to other reports on AgNPs. Interestingly in our study the MIC and MBC for gram-positive bacteria i.e. *S. epidermidis* was lesser than *E. coli.* On the other hand, the ZOI was greater for *S. epidermidis* which indicates that *S. epidermidis* is more sensitive to SpNPs regardless of its membrane thickness which normally resists the penetration of AgNPs^7,60^. Therefore, we anticipate SpNPs will be more effective against multidrug-resistant strains of gram-positive bacteria such as *S. aureus*, etc. The exact mechanism for the antimicrobial activity of AgNPs is still being extensively studied and mostly attributed but not limited to cell membrane penetrations, reactive oxygen species production and ribosome disassembly^4,9^. Our observational study of nuclear staining of bacterial cells using Syto 16 provide insights on the killing mechanism of SpNPs which seemed to be the degradation of bacterial cell membranes as demonstrated by using a membrane-permeable fluorescent dye. We observed fluorescence in untreated bacterial cells whereas, SpNPs treated bacterial cells produced no observable fluorescence at ≥ 100 µg/mL after 4 h.

Our synthesized SpNPs may have broad applications in biomedical field, for example they can be used for wound healing^61^, as anticoagulant, anticancer, and for anti-inflammatory biosensing applications^36^. Moreover, SpNPs may also have environmental applications^15^ to reduce contaminants such as microbial pathogens and toxic compounds including dyes and heavy metals. The entrapment of SpNPs in porous polymeric or cellulosic materials can also be employed for water treatment point of use devices. Similarly, SpNPs can be used for wound healing if combined with other supportive materials such as hydrogels and or cotton materials etc. Moreover, lower MIC value of our SpNPs will be useful to overcome the problem of cytotoxicity in water purification applications. However, a key requirement of using SpNPs for the human centered applications is to study their in-vivo toxicity to understand the time and dose dependent cytotoxicity of SpNPs.

## Conclusions

We reported an eco-friendly and simpler method for the synthesis of more effective silver nanoparticles using aqueous extract of *S. persica* roots. The analysis of UV–Vis, TEM, FE-SEM, DLS, FTIR and P-XRD demonstrated the presence of spherical and rod shaped SpNPs with an average size of 37.5 nm, presence of phytochemical head groups, and crystallographic structure. These NPs have shown a high efficacy of antimicrobial activity against *S. epidermidis* and *E. coli* bacteria at < 1 µg/mL (MIC) as compared to earlier reports. Furthermore, we observed the killing mechanism of SpNPs seems to be the degradation of bacterial cell membranes upon interaction and incubation as demonstrated by using a membrane-permeable fluorescent dye. Our SpNPs have shown significantly higher effectiveness against bacterial pathogens which depicts their potential use for many antimicrobial applications in the future.

## Supplementary information

Supplementary information.

## Abbreviations

AgNPs: Silver nanoparticles
Sp: *Salvadora persica*
FTIR: Fourier transform infrared spectroscopy
TEM: Transmission electron microscopy
FE-SEM: Field emission scanning electron microscopy
DLS: Dynamic light scattering
XRD: X-ray powder diffraction
MIC: Minimum inhibitory concentration
MBC: Minimum bactericidal concentration
