# Supplementary material for: Salvadora persica mediated synthesis of silver nanoparticles and their antimicrobial efficacy
Source: Sci Rep. 2021 Mar 16;11:5996. doi: 10.1038/s41598-021-85584-w (PMC7966387; doi:10.1038/s41598-021-85584-w)
Supplement: Supplementary file 1 — Supplementary information. [file 41598_2021_85584_MOESM1_ESM.docx]

**Supplementary Information**

***Salvadora persica* Mediated Synthesis of Silver Nanoparticles and their Antimicrobial Efficacy**

Hammad Arshad^1,2,4^, Muhammad A. Sami^2^, Saima Sadaf^1^, Umer Hassan*^2,3^

^1^ Institute of Biochemistry & Biotechnology, University of the Punjab, Lahore. Pakistan

^2^ Department of Electrical and Computer Engineering, School of Engineering, Rutgers The State University of New Jersey, Piscataway, NJ, USA

^3^ Global Health Institute, Rutgers The State University of New Jersey, New Brunswick, NJ, USA

^4^ Department of Biology, Lahore Garrison University, Lahore, Pakistan

*Corresponding Author: umer.hassan@rutgers.edu; Tel (848) 445-2164

**Table S1.** The optimization of SpNPs synthesis at room temperature. The variables are shown in the table; highlighted ratios were further subjected to optimize time.

| Sr. No. | Solution X  (mM) | Solution Y  (w/v) | Ratio X:Y  (10ml) | Solution Z  color change | Incubation time (h) |
| --- | --- | --- | --- | --- | --- |
| 1 | 1 | 1% | 9:1 | Nil | 96 |
| 2 | 2 | 1% | 9:1 | Nil | 96 |
| 3 | 5 | 1% | 9:1 | Nil | 96 |
| 4 | 10 | 1% | 9:1 | Nil | 96 |
| 5 | 1 | 5% | 9:1 | Nil | 96 |
| 6 | 2 | 5% | 9:1 | Nil | 96 |
| 7 | 5 | 5% | 9:1 | Nil | 96 |
| 8 | 10 | 5% | 9:1 | Slightly yellow | 96 |
| 9 | 1 | 10% | 9:1 | Nil | 96 |
| 10 | 2 | 10% | 9:1 | Light yellow | 96 |
| 11 | 5 | 10% | 9:1 | Light yellow | 96 |
| 12 | 10 | 10% | 9:1 | Yellow | 96 |


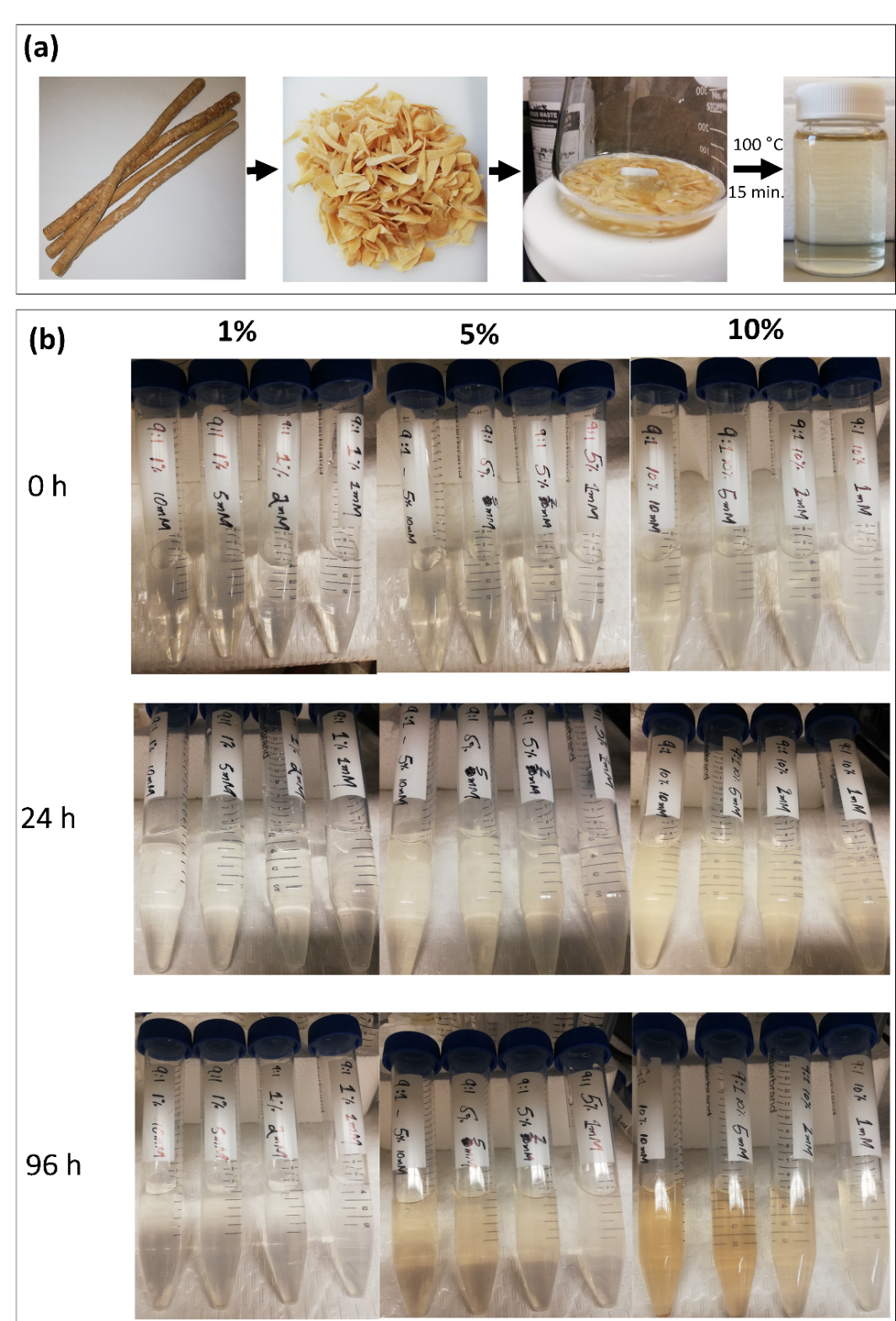


**Fig. S1.** The aqueous extraction of *Salvadora persica* was carried out in distilled H_2_O as shown in (a). The optimization for synthesis at room temperature in shown in (b), 1%, 5% and 10% of solution Y was mixed with 1mM, 2 mM, 5 mM and 10 mM of solution X in a fixed ratio of 1:9. The change in color was noticed and proceeded accordingly for further optimizations.


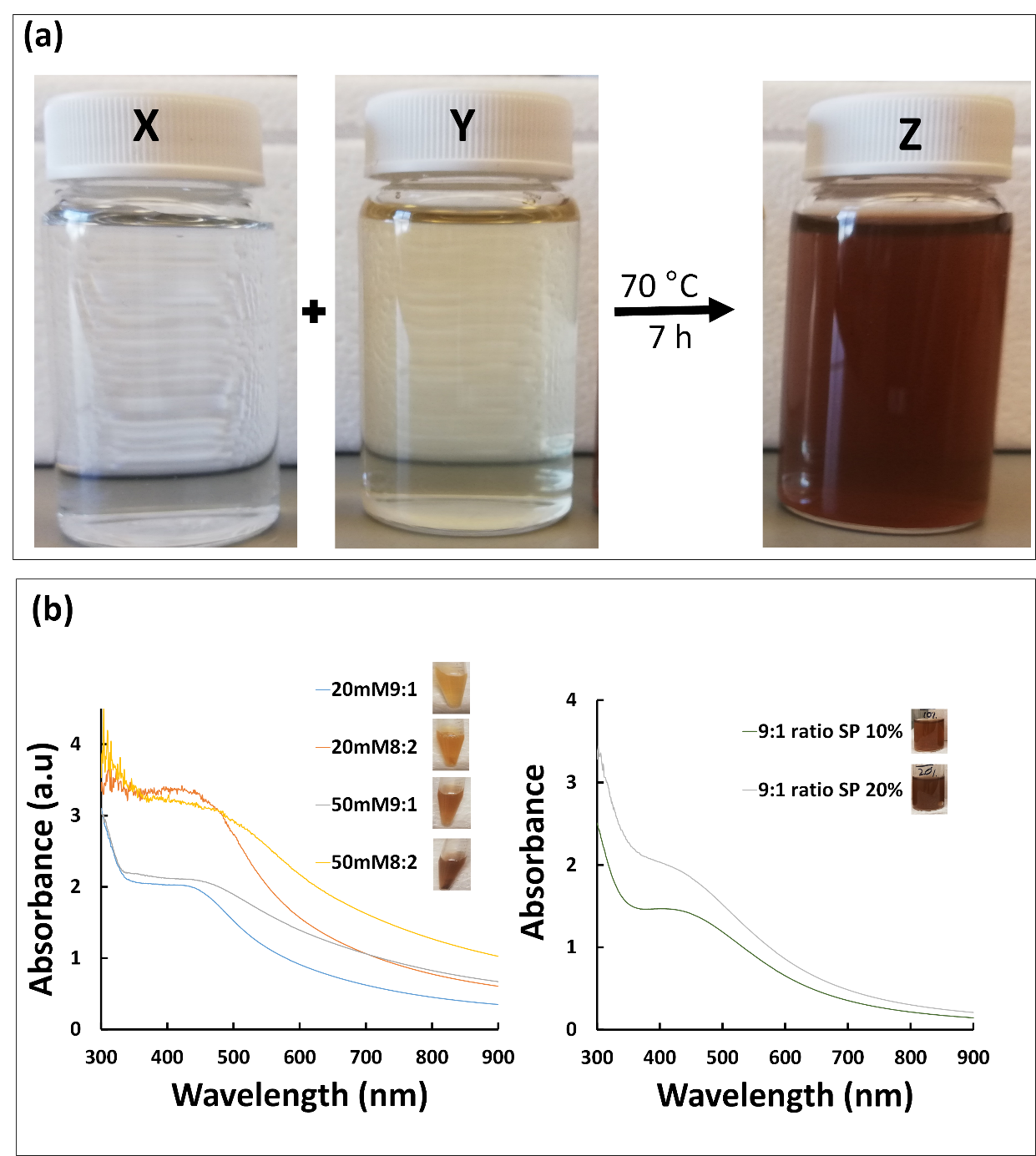


**Fig. S2**. Synthesis of silver nanoparticles, where solutions X and Y were mixed in 9:1 and the color change were clearly observed after incubation at 70 °C as shown in (a). Higher concentration of solution X and solution Y also showed the presence of SpNPs but with agglomerations as depicted in UV-Vis analysis. 10% solution Y with 10 mM of solution X produced best spectrum (b).


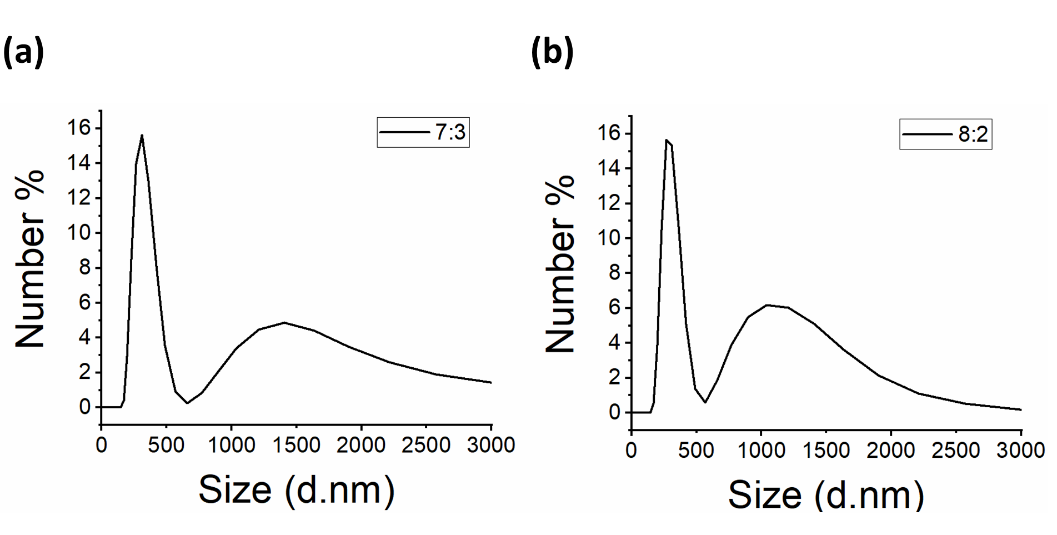


**Fig. S3**. Dynamic Light Scattering. The effect of higher ratio of solution Y to solution X represented the rise in size and agglomeration of SpNPs as shown in (a) and (b). The analysis suggested the use of lower ratio (9:1) of solution Y.
